# Impacts of COVID-19 on trans and non-binary people in Canada: a qualitative analysis of responses to a national survey

**DOI:** 10.1186/s12889-022-13684-x

**Published:** 2022-07-02

**Authors:** Hannah Kia, Leo Rutherford, Randy Jackson, Alisa Grigorovich, Carol Lopez Ricote, Ayden I. Scheim, Greta R. Bauer

**Affiliations:** 1 School of Social Work, The University of British Columbia, 2080 West Mall, Vancouver, BC V6T 1Z2 Canada; 2 School of Public Health and Social Policy, University of Victoria, Room B202, Human and Social Development Building, 3800 Finnerty Road, Victoria, BC V8P 5C2 Canada; 3 School of Social Work, McMaster University, Room 319, Kenneth Taylor Hall, 1280 Main Street West, Hamilton, ON L8S 4M4 Canada; 4 Department of Health, Aging and Society, McMaster University, Room 226, Kenneth Taylor Hall, 1280 Main Street West, Hamilton, ON L8S 4M4 Canada; 5 Recreation and Leisure Studies, Brock University, 1812 Sir Isaac Brock Way, St Catharines, ON L2S 3A1 Canada; 6 Department of Epidemiology and Biostatistics, Western University, 3rd Floor, Western Centre for Public Health and Family Medicine, 1465 Richmond Street, London, ON N6G 2M1 Canada; 7 Department of Epidemiology and Biostatistics, Dornsife School of Public Health, Drexel University, Nesbitt Hall, 3215 Market Street, Philadelphia, PA 19104 USA

**Keywords:** Transgender, Non-binary, COVID-19 impacts, Qualitative research, Thematic analysis, Canada

## Abstract

**Background:**

Emerging international evidence indicates the COVID-19 pandemic has exacerbated socioeconomic and health challenges faced by transgender (trans) and non-binary populations globally. This qualitative study is among the first to characterize impacts of the pandemic on these groups in Canada.

**Methods:**

Drawing on data from the Trans PULSE Canada survey (*N* = 820), we used thematic analysis to examine the free-form responses of 697 participants to one open-ended question on impacts of the pandemic. We first organized responses into descriptive themes, and then used this preliminary analytical process to construct more refined, higher order themes that provided a rich account of the pandemic’s impacts.

**Results:**

Our results are organized into five themes that highlight the pandemic’s impacts on trans and non-binary populations in Canada. These include: (1) reduced access to both gender-affirming and other healthcare, (2) heightened financial, employment, and housing precarity, (3) strained social networks in an era of physical distancing and virtual communication, (4) an intensification of safety concerns, and (5) changes in experiences of gender affirmation.

**Conclusion:**

Our findings highlight the pandemic’s systemic impacts on the lives of trans and non-binary people in domains such as healthcare, employment, and housing, and on the social networks of these groups, many of which reflect an exacerbation of pre-existing inequities. Based on our analysis, we recommend that public health researchers, policymakers, and practitioners attend to the structural impacts of the pandemic on these groups as primary sites of inquiry and intervention.

## Background

Since the start of the COVID-19 pandemic, a growing body of literature has highlighted the ways in which the virus has impacted populations beyond illness and mortality. A few of these cross-population burdens have included social isolation [1–3], stress and anxiety [4–10], reduced healthcare access [11–14], as well as loss of income and financial strain [15]. Certain marginalized groups, particularly those reporting profound social and health inequities since before the COVID-19 era, may be disproportionately affected by the pandemic [16]. Transgender (trans) and non-binary people, who are often exposed to high levels of stigma, discrimination, and other adverse structural conditions, and frequently report significant disparities in connection with these experiences [17, 18], may be among these populations. Indeed, trans and non-binary people’s high pre-pandemic burdens of poor mental health, profound barriers to healthcare access, and adverse socioeconomic conditions have long been documented [6, 7] and have recently been highlighted to substantiate the need for research on the issues of these communities in the COVID-19 context [16, 19].

The few empirical studies published to date, primarily from non-Canadian jurisdictions, have documented unique impacts of the pandemic and its related public health directives on trans and non-binary people. The emerging literature has highlighted, at a preliminary level, social isolation and declining mental health [16, 20], reduced access to gender-affirming care such as hormones and surgeries [21, 22], as well as an overall exacerbation of the social and economic marginalization of trans communities [23, 24], as representing some of the most salient impacts of the pandemic for these populations thus far. Of note, some of these impacts appear to be intricately interrelated. For example, the reduced access of trans and non-binary people to gender-affirming care has been associated with increases in stress, suicidality, and overall poorer self-reported mental health [22, 23].

Little of the emerging literature in this area has been specific to the Canadian context. One quantitative study has substantiated the potentially negative mental health impacts of the COVID-19 pandemic on trans and non-binary communities in Canada [20]. In this study, to assess mental health, Hawke and colleagues incorporated the CoRonavIruS Health Impact Survey (CRISIS), which was utilized to compute a mean score representing the mental health impacts of the pandemic based on participants’ self-reporting of conditions such as worry, irritability, and unmet mental health needs. To highlight some findings of this study, the data revealed that in the context of the COVID-19 pandemic, trans and gender diverse youth experienced a larger burden of mental health and substance use issues, and greater declines in social support from their families, relative to their cisgender counterparts. Another study, which indicated that 52.9% of trans and non-binary participants were unable to meet financial obligations or pay for essential needs (such as housing, utilities, and groceries) as a result of the COVID-19 pandemic, has contributed evidence on the worsening of socioeconomic conditions among these populations in Canada [25] . These issues have, indeed, been documented despite the presence of government assistance during the pandemic through the Canada COVID-19 Economic Response Plan [26], along with comparable initiatives at provincial and territorial levels of government [27], and a pre-pandemic recognition of the need to address the socioeconomic precarity of LGBTQ2 populations in Canada’s Poverty Reduction Strategy [28].

Together, existing studies indicate the relevance of further developing the body of research concerned with the COVID-19 pandemic’s impacts on trans and non-binary populations in Canada. Qualitative studies can be critical in contextualizing and generating insight on under-examined social conditions and experiences that help explain the health inequities of marginalized populations, particularly those that have been exacerbated in the era of the COVID-19 pandemic [29]. Given recent calls for a deepening [9] and enhanced contextualization [22] of the existing knowledge of the impacts of the COVID-19 pandemic on trans and non-binary communities in Canada and elsewhere, such inquiry could help explain what appear to be worsening disparities for gender minorities. In this qualitative study, we address the gaps in the literature by exploring the impacts of the COVID-19 pandemic on trans and non-binary people in Canada.

## Methods

This paper is based on a qualitative analysis of participants’ free-form responses to one open-ended question (“Can you tell us how the COVID-19 pandemic has impacted you as a trans or non-binary person, whether positive or negative?”) on a national survey examining the impacts of the COVID-19 pandemic on trans and non-binary people in Canada. Though surveys are often primarily considered appropriate for quantitative studies, some have discussed the suitability of applying qualitative methods (described below) to analyze free-form responses on survey instruments designed to address under-examined questions requiring a more inductive approach [30, 31].

### Data collection

Between September and October 2020, we conducted a national online survey of 820 trans and non-binary people across Canada to investigate the impacts of the COVID-19 pandemic on these populations. This study was a follow-up to Trans PULSE Canada, a national community-based survey on the health and well-being of trans and non-binary people, which involved a 10-week period of data collection in 2019. Eligibility criteria for the original survey included identifying with a gender different from the one assigned at birth, being at least 14 years of age, and living in Canada. To participate in the follow-up survey, individuals must have completed the baseline survey and have continued living in Canada. The parent survey, as with the current follow-up study, involved extensive collaboration with a large national network of community partners and researchers to develop and execute recruitment and sampling methods, along with data collection instruments and procedures, which together reflected sensitivity to the social context of trans and non-binary people in Canada. This approach represented the leveraging of community-based approaches for strengthening community relevance and scientific rigour in data collection involving marginalized populations [32]. We recruited 2873 respondents into our parent study, of whom 1184 provided consent for recruitment in follow-up studies, including the current COVID-19 survey. In this article, we examine the responses that 697 participants (of the total 820 COVID-19 survey respondents) gave to one of the open-ended questions inviting them to broadly reflect on how they have been impacted by the COVID-19 pandemic.

### Data analysis

Qualitative analyses of survey responses are compelling in situations involving the study of under-explored issues and experiences among highly heterogeneous populations. This is because they involve investigating free-form participant responses whose contents researchers may not have anticipated in the design of fixed survey response items typically used in quantitative analyses, and because (especially in large samples) they can yield a broad range of responses [30]. Given that little is known about the impacts of COVID-19 on trans and non-binary populations in Canada [18], and that these groups tend to be highly heterogeneous in relation to factors such as gender, race, socioeconomic status, and other variables [20], our analytic methods for examining substantial free-form survey responses align well with the scope and aims of this study. Although almost all of our analysis was qualitative, to account for selection bias, we ran chi-square tests to compare the demographic characteristics of the 697 who provided free-form responses to this question against the 123 who did not. We found no statistically significant differences between these groups.

Consistent with survey-appropriate approaches to thematic analysis [30], our analytical process involved an inductive and iterative process of sorting and defining the data, developing categories, and identifying more refined and interpretive higher order themes. Specifically, based on readings of the entire dataset, the lead author (HK) prepared a draft conceptualization of key descriptive themes reflected across participant accounts, which she then shared with the team for feedback. All of the team members then met and discussed these codes and the development of broader categories. The lead author subsequently used input from the team to transform the descriptive categories into more analytically consolidated themes to construct an overarching account of the COVID-19 pandemic’s impacts on trans and non-binary people in Canada, including those in domains such as healthcare, housing, employment, and social networks. Of note, qualitative analyses of survey data can be either descriptive and/or interpretive, depending on the scope and aims of a study [30, 33, 34]. In this study, our aim was primarily to describe the range of the pandemic’s impacts on trans and non-binary populations, but we engaged in a preliminary interpretation of the data to consolidate a larger number of descriptive categories into a smaller number of higher order themes. While the lead author led this step to enhance the intelligibility of our findings, she incorporated feedback from all members of the authorship team during this process to ensure refined interpretations of the data continued representing the breadth of participant accounts. In particular, consistent with thematic analysis [33], the lead author invited other members of the authorship team to provide feedback on the fidelity of higher order themes to preliminary descriptive categories, and incorporated this feedback to enhance analytical rigour. Additionally, to ensure the trustworthiness of the findings, a draft of the manuscript (which included contents of the final analysis) underwent review by the Steering Committee of the Trans PULSE Study, whose membership comprises a majority of trans persons and includes representation from community stakeholders. The team incorporated feedback from the Steering Committee to strengthen the team’s final conceptualization of the findings.

The lead author, together with the wider authorship team, drew on an intersectional lens [35, 36] to inform the presentation of the data by ensuring participant quotes selected for this article were contextualized with information about each participant’s gender, race, age, and any other relevant features of their social location. The team also used intersectionality in selecting quotes with the greatest potential to foreground the rich diversity of experiences reflected along joint axes of difference such as gender and race and, in so doing, ensure explicit representation from across a heterogeneous sample of trans and non-binary people whose experiences are shaped by the ways that social power plays out across intersections. Our analysis seeks to incorporate consideration of social intersectional location to inform an understanding based in intersectionality’s core tenets, including social power, intersecting power relations, social context, relationality, complexity, and social justice [36]. In this study, we do this by foregrounding the diversity of the accounts of participants located at different axes of marginalization, but as we note in the discussion, our descriptive analysis is limited in its capacity to inform substantive inferences about the role of intersecting oppression in shaping experience, and we believe more conceptual inquiry to generate such insight is warranted.

### Compliance with ethical standards

This study underwent review and received approval from Western University’s Non-Medical Research Ethics Board (NMREB). All respondents provided informed consent to participate in this study. Consent for publication of quotes was provided separately by 814 of 820 participants; for the other six participants, entries were used for overall interpretation, but without incorporation of individual quotes. As trans and non-binary youth are often at a significant risk of harm if they reveal their gender identity to potentially unsupportive or hostile parents or guardians, all participants under the age of majority self-consented to participate. This was a measure that was approved by Western University’s NMREB.

## Results

Of the 820 respondents to our follow-up survey, the majority were under 35 (*n* = 516, 63%, age range: 15–75). Of respondents who were 25 years of age or older, most were employed (*n* = 428, 72%) and reported an annual income of under CAD $30,000 (*n* = 350, 58%). For context, an annual income of CAD $30,000 is considerably lower than the 2019 median income of Canadians ages 25–54 ($48,200) and those ages 55–64 ($42,800), but comparable with the median income of Canadians ages 65+ ($30,400) [37]. Approximately 13% (*n* = 108) of respondents in our sample were racialized, meaning they indicated either identifying accordingly or being “perceived or treated as a person of colour in Canada,” and 7% (*n* = 59) self-identified as Indigenous. With regard to gender, 25% (*n* = 201) identified as girls/women, 24% (*n* = 200) as boys/men, 49% (*n* = 400) as non-binary, and 2% (*n* = 18) identified with Indigenous or culturally-specific genders. A small minority of participants reported residing in rural regions of Canada with populations of less than 10,000 (*n* = 48, 6%). We provide an overview of the respondents’ demographic characteristics in Table 1, stratified by whether they provided free-form responses.

**Table 1:** Characteristics of Trans PULSE Canada COVID Cohort participants

	Total sample ***n*** = 820	Completed free-form responses to the question: “Can you tell us how the COVID-19 pandemic has impacted you as a trans or non-binary person, whether positive or negative?		
		Yes ***n*** = 697	No ***n*** = 123	***p***-value^**a**^
	n (%)	n (%)	n (%)	
**Age**				0.767
15–19	57 (7)	46 (7)	11 (9)	
20–24	151 (19)	132 (19)	19 (16)	
25–34	308 (38)	261 (38)	47 (39)	
35–49	226 (28)	190 (27)	36 (30)	
50–64	62 (8)	55 (8)	7 (6)	
65 +	10 (1)	9 (1)	1 (0.8)	
**Gender**				0.620
Woman or girl	201 (25)	176 (25)	25 (20)	
Man or boy	200 (24)	167 (24)	33 (27)	
Indigenous or cultural gender	18 (2)	16 (2)	2 (2)	
Non-binary or similar	400 (49)	337 (48)	63 (51)	
**Indigenous in Canada**				0.965
Indigenous in Canada	59 (7)	50 (7)	9 (7)	
Not Indigenous in Canada	758 (93)	644 (93)	114 (93)	
**Racialization**^**b**^				0.277
Racialized	108 (13)	88 (13)	20 (16)	
Not racialized	710 (87)	607 (87)	103 (84)	
**Urban / rural**^**c**^				0.366
Rural or small town	48 (6)	43 (6)	5 (4)	
Not rural or small town	769 (94)	652 (94)	117 (96)	
**Employment (age ≥ 25)**^**d**^				0.377
Permanent full-time	220 (37)	184 (36)	36 (40)	
Employed, not permanent full-time	208 (35)	176 (35)	32 (36)	
Not employed or on leave	137 (23)	122 (24)	15 (17)	
Not employed and student or retired	33 (6)	26 (5)	7 (8)	
**Past-year personal income (age ≥ 25)**^**d**^				0.292
None	67 (11)	53 (10)	14 (15)	
< $15,000	149 (25)	132 (26)	17 (19)	
$15,000 - $29,999	134 (22)	116 (23)	18 (20)	
$30,000 - $49,999	115 (19)	99 (19)	16 (18)	
$50,000 - $79,999	91 (15)	72 (14)	19 (21)	
$80,000 +	48 (8)	41 (8)	7 (8)	

^a^*P*-values obtained from chi-square tests

^b^Racialized defined as either identifying as a person of colour or indicating they were perceived or treated as a person of colour

^c^Rural or small town defined as living in a community with population of less than 10,000

^d^Employment and economic questions were asked for those age 25 and older, as participants could be as young as 14 years of age

Our results are organized into five themes, which together reflect the range of impacts of the COVID-19 pandemic on trans and non-binary people involved in our national survey. These include: (1) diminished access to healthcare, including gender-affirming care, (2) an exacerbation of financial, employment, and housing precarity, (3) impacts of physical distancing and virtual communication on social networks, (4) an increase in safety concerns, and (5) changes in experiences of gender affirmation.

### Diminished access to healthcare, including gender-affirming care

Much of our data illustrated compromised access to healthcare among trans and non-binary people in Canada due to the COVID-19 pandemic. Although these populations already experienced profound barriers to healthcare access prior to COVID-19 [18], the pandemic appears to have exacerbated this problem. For example, one Indigenous participant discussed an increasingly limited network of systems providing in-person healthcare in the COVID-19 era, together with an absence of trans-inclusive healthcare providers within some of these sparse settings, as negatively impacting quality and access to appropriate care:

I’ve had to spend three hours going across the city for medical care, only to be called slurs in the washroom there and misgendered by the professionals there, for care I couldn't receive there. It's been exhausting and lonely, and I don't see it improving anytime soon. (Age 25-34, Indigenous, in the Prairies)

It is important to note that the participant above could have either been referring to general healthcare or gender-affirming care in their account. Although many participants discussed general reductions in healthcare access, others specifically highlighted increased barriers to gender-affirming medical care. Among these participants, the most significant concerns appeared to be the rescheduling of surgeries, the unavailability of hormones, and the scarcity of specialist care sometimes required for the provision of gender-affirming services. For example, one participant indicated:

My greatest concern as a trans person during COVID-19 is the huge backlog of 'non-essential surgeries' (i.e., gender-affirming surgeries) that is destined to occur. I also worry about how my access to hormones will be affected. (Age 20-24, white, in British Columbia [BC])

Together these two respondent accounts revealed the prominent impacts of the COVID-19 pandemic in limiting the availability of healthcare options for trans and non-binary people in Canada and, in many cases, access to gender-affirming care for these populations. Given that both respondents quoted here reported living in regions with populations of over 10,000, and that participants located in less populated rural environments provided similar responses, these problems appeared to be salient across regions with varying levels of population density.

Despite problems with access to care, including disruptions in the provision of general and gender-affirming healthcare, some participants reported avoiding interruptions to their healthcare by receiving services virtually and, in rare cases, improvements in accessibility as a result of access to virtual care. Experiences with virtual care were mixed . Some respondents, for example, described poorer quality care as a result of what they perceived to be the reduced availability of healthcare providers with knowledge of gender-affirming care in this context. However, a few discussed experiencing fewer barriers to care than before the pandemic (particularly those related to distance, transportation, and accessibility) because of this shift. For example, one respondent (age 20–24, white, in the Prairies), highlighted that for them, access to gender-affirming healthcare had continued and in fact improved because of the transition to virtual care: “the majority of my healthcare (particularly gender-affirming healthcare) has gone uninterrupted, having simply moved to virtual healthcare, which is more accessible for me.” Participant accounts such as these may have been reflective of respondents’ resistance to interruptions in healthcare brought on by the pandemic.

### An exacerbation of financial, employment, and housing precarity

Not surprisingly, and despite Canada’s COVID-19 Economic Response Plan, respondents frequently discussed increased economic insecurity as a prominent impact of the COVID-19 pandemic. Importantly, participants who highlighted financial strain commonly described increasingly limited options for safe and well-compensated employment among trans and non-binary people specifically. Given the pre-pandemic ubiquity of discrimination against trans and non-binary people in Canada [18], notions of ‘safe employment’ in many participant accounts may have referred to work contexts with less perceived hostility to these groups [38]. Illustrating the potentially increased insecurity of the COVID-19 labour market for trans and non-binary people in Canada, one racialized (mixed Southeast Asian and white) non-binary participant (age 25–34, in BC) provided the following account: “it’s mostly on the job front, I worry that it’ll be more difficult for me to get something that pays me appropriately, that I feel safe doing, as a trans person.” Relatedly, participants who had access to paid work prior to the pandemic also commonly described losing their employment. For example, one Alberta-based respondent (white, age 20–24) indicated experiencing the unexpected loss of an employment contract: “My contract for my position as an LGBTQ+ educator and mentor was also terminated early.”

Aside from indicating declines in their general financial stability, participants also commonly highlighted worsening housing precarity in their accounts of the pandemic’s impacts on their health and well-being. For example, one youth respondent, who was also racialized and non-binary, remarked on the negative mental and physical health impacts of housing insecurity:

I have had several periods of housing instability. This has negatively impacted my mental and physical health to the point where I'm seeking access to the necessary resources. (Age 20–24, Black Caribbean, in BC)

In addition to general comments concerning housing precarity, participants commonly described having to make unanticipated, yet impactful decisions related to their living arrangements, particularly considering physical distancing measures and/or unforeseen financial problems brought on by the labour market, which has been limited due to the pandemic. Some respondents discussed having to temporarily move in with their families of origin, many of whom were hostile to trans and non-binary people, and consequently indicated experiencing significant declines in their mental health. Others described positive experiences of moving in with friends and partners, which yielded unexpectedly favourable impacts on their mental health and reflected resilience in the context of the pandemic. One youth participant described having to stay with unsupportive parents at the start of the pandemic, and then moving twice to eventually live alone. Despite the hardship brought on by this experience, the respondent described feeling grateful for the presence of a supportive trans community during these transitions:

I had planned to move out of my parents' home in March. This was delayed, and I ended up living with them until mid-July. I became suicidal and moved in with friends for a couple of months. I have just moved again now, and am living alone … I [feel] incredibly grateful for the ways my trans community has shown up for me. (Age 15-19, Jewish and white, in Ontario)

### Impacts of physical distancing and virtual communication on social networks

Participants regularly commented on the variable impacts of physical distancing and the shift to virtual communication on their social networks. Some respondents specifically mentioned the fragility of their pre-pandemic relationships and highlighted the role of physical distancing in compromising these tenuous social ties and worsening feelings of isolation. One respondent, for example, discussed the near absence of contact with others in the context of physical distancing:

My social circles are gone. I'm able to touch or be intimate with only one person. Attempts to maintain social contact of any kind are almost entirely blocked. Being trans and NB [non-binary] means that social circles are very small, and now they are mostly gone. (Age 50-64, white, in BC)

Although participants often discussed the use of virtual platforms to connect with others socially, some described the challenges of maintaining ties to trans and non-binary communities when they were exclusively able to do so online. One racialized youth respondent, for example, highlighted the negative impact of losing access to in-person LGBTQ+ groups. This respondent also discussed the difficulty of using alternative virtual spaces while staying with family, and lacking the privacy needed in this setting to connect with community members online. Further complicating the transition to virtual communication, this individual also indicated being part of a social network that consisted of people with limited social media presence:

The greatest loss is the loss of in-person LGBTQ+ groups … There are a few online groups still going, but I’m just not comfortable meeting people online in my own house the way I was going out in person where my parents aren’t around (they’re not unsupportive, but I’m still wary). Furthermore, some of the people who I bonded with just don’t have social media or aren’t open on it. (Age 20-24, East Asian, in Ontario)

It is important to note that some participants discussed the transition to virtual communication favourably. One non-binary respondent, for example, who described starting an online queer community during the pandemic, noted that the group enabled this individual to “come out” to this community and experience validation from its members:

In my desire to make sure that younger queer people feel supported and less isolated when stuck at home with homophobic/queerphobic [parents], I started an online community … and through that I've become a lot closer with people who affirm my gender identity. I wasn't out to anyone in real life before the pandemic, but having the community online has made me feel validated, and I’ve come out to a few people in person. (Age 25-34, white, in Atlantic Canada)

As reflected in these narratives, some of which may have represented expressions of collective resilience, the transition to virtual communication was not entirely negative among our participants, but did represent a significant shift in experiences of developing and sustaining social networks. Given the precarity of the pre-existing social networks of some trans and non-binary people, particularly those with barriers to means of connecting with peers [39], these variable experiences are (regardless of their nature) important to highlight as being reflective of the profound impacts of the pandemic.

### An increase in safety concerns

Participants regularly reported an increase in their safety concerns during the COVID-19 pandemic. Many respondents indicated that measures such as physical distancing or adhering to stay-at-home orders had limited their capacity to be in the presence of other trans and non-binary people in public spaces, and that they had experienced a heightened susceptibility to public scrutiny, violence, and victimization while navigating these contexts alone or in smaller numbers. Illustrating the complexity of this phenomenon, one non-binary participant indicated being in a relationship and experiencing scrutiny in public spaces for being perceived to socialize with an individual outside the nuclear family structure:

During the early days of the pandemic, I was aware of being more visible as a queer person in a relationship that perhaps appeared to be outside of nuclear family and therefore breaking [physical distancing] guidelines. Also, more disconnected from queer and trans communities. (Age 20-24, white, in BC)

Several participants also highlighted what they perceived to be overall declines in the mental health of the general population, which they believed exacerbated their pre-pandemic vulnerability to anti-trans hostility. One respondent (age 35–49, white, in the Prairies), for example, mentioned: “I think it’s [the pandemic] made people more angry and short-fused and they’re looking for easy targets to take it out on, like us.”

### Changes in experiences of gender affirmation

Finally, participant accounts frequently highlighted changes that trans and non-binary people experienced in relation to seeking and receiving social affirmation for their gender. Some respondents discussed diminished opportunities for publicly expressing their gender, given prolonged periods of isolation and the use of face-covering masks while in public. While some articulated feeling distressed over this change, as they experienced fewer opportunities for others to validate their gender, others described feeling relief over being less ‘visibly’ trans or non-binary in potentially trans-antagonistic public spaces. One respondent, for example, indicated having both experiences:

It’s weird because being seen and how I present myself is both anxiety filled and also necessary. Like, when COVID happened the fact that I wouldn't be dressing up for work bothered me. On the other hand, I still do my makeup [every day] because of Zoom meetings, so there's some continuity … [also] having to wear a mask in public can be really, really, wonderful because I don't need to worry about being clocked [recognized as trans] as much. (Age 35-49, white, in the Prairies)

Interestingly, several respondents discussed using the relative ‘safety’ of their time in isolation to explore their gender expression and gender identity more fully than before the pandemic, in turn revealing an underlying resilience in the context of fewer opportunities for public forms of affirmation. In general, these accounts reflected respondents’ leveraging of public health measures such as physical distancing to privately explore means of experiencing gender affirmation while in isolation. Illustrating this theme, one racialized and non-binary participant (age 25–34, East Asian and Southeast Asian, in Ontario) wrote: “it’s given me more time to think about how I present or how I want to present without having to be hyper aware of my environment.” Drawing on these comments, although pandemic-related changes in social gender affirmation were salient for participants, these shifts were variably experienced as limiting for some, and as helpful for others who felt able and comfortable to use their time in isolation to explore their gender at home.

## Discussion

In a Canadian study exploring the impacts of COVID-19 on trans and non-binary populations, and specifically, drawing on qualitative responses to a national survey, we found that the pandemic may have: (1) increased barriers to healthcare (including trans-affirming care), (2) exacerbated socioeconomic precarity, (3) impacted social networks by way of physical distancing and the shift to virtual communication, (4) heightened safety concerns, and (5) altered experiences of gender affirmation. In general, the insights reflected in our research are congruent with many of the issues highlighted in the sparse international scholarship on trans and non-binary people’s experiences with COVID-19. For example, previous work in this area has substantiated the salience of reduced access to healthcare [21, 22], the growing use of virtual and other remote care options [40], as well as the worsening of pre-existing socioeconomic disparities [23, 24, 41]. Some of the other emerging scholarship in the Canadian context has similarly illustrated the impacts of the pandemic on the capacity of trans and non-binary people to meet financial or essential needs, to live in supportive environments, and to maintain their health and social contact with peer communities [25]. Our work strengthens this scholarship by further substantiating the relevance of these issues for the Canadian context, and by shedding light on the high level of variation in healthcare, socioeconomic, and social support experiences of trans and non-binary people in the COVID-19 context.

Importantly, our study contributes complexity to the existing literature by yielding novel, nuanced insights on how the COVID-19 pandemic has impacted trans and non-binary communities. Foremost, although isolation and poor mental health have previously been found to represent some of the pandemic’s most significant impacts on these groups [16, 20], our findings reveal that declines in mental health may be secondary to the systemic impacts of public health restrictions on the healthcare access, socioeconomic stability, general safety, and community connectedness of trans and non-binary people. Indeed, although our participants commonly discussed experiencing isolation, anxiety, distress, and suicidality, they most often described these experiences in connection with specific healthcare barriers, experiences of socioeconomic instability, strained social networks, safety issues, and fewer social opportunities to feel affirmed in their gender, and rarely mentioned their mental health without also referring to these other issues. We have intentionally remained descriptive in our analysis, given the paucity of existing research in this area and the relevance of starting with description in thematic analyses involving under-examined phenomena [33]. However, our utilization of intersectionality [35] to contextualize participants’ quotes reveals that individuals involved in the study were highly diverse in relation to social location, and may have experienced the aforementioned structural problems in the course of having to navigate interlocking systems of oppressions. More conceptually driven qualitative research will be needed in the future to identify how oppressive forces such as transphobia, racism, ageism, and classism specifically intersect to shape the COVID-19 pandemic’s systemic impacts on trans and non-binary populations.

Second, while our findings suggest that trans and non-binary people may be disproportionately burdened by public health restrictions brought on by the COVID-19 pandemic [22, 24, 25], they also highlight a limited array of positive experiences that demonstrate the strength and resilience of trans and non-binary people in this context. Some of our respondents, for example, discussed encountering enhanced accessibility to gender-affirming care in virtual contexts, an opportunity to cohabitate with supportive friends and partners and build affirming virtual communities, and an increased capacity to privately explore their gender during periods of isolation. Finally, although there does exist preliminary insight on the role of the COVID-19 pandemic in exacerbating economic insecurity for trans and non-binary people in Canada [25] despite the availability of government-administered financial support, our study sheds light on specific forms of socioeconomic instability that may be particularly prominent. For example, our work foregrounds the limited options for safe and accessible employment – and the salience of unemployment – among trans and gender non-binary people in the COVID-19 context, and additionally draws attention to the centrality of housing instability and its impacts on mental health.

Our study has several limitations. Most importantly, as this is a qualitative analysis of free-form responses provided on a survey, we acknowledge that we were unable to incorporate certain measures typically used by qualitative researchers to enhance rigour in research design. First, we were not able to verify our initial understanding of participant experiences using live prompts, which is common practice in the collection of qualitative interview data [34], and typically used to enhance the fidelity of researchers’ interpretations of the data to meanings originally intended by participants. Relatedly, while we did include several open-ended questions in our survey, this study focused exclusively on responses to one question to which respondents provided particularly detailed and rich responses. This is because participants were less likely to respond to other open-ended questions, and often wrote briefer responses to these other prompts that were not well suited to thematic analysis. Although it is acceptable for qualitative analyses of survey data to be based on responses to a single item – particularly one that yields significantly richer data than other items [30] – the quality of textual data (and thus the depth of the resulting analysis) can greatly improve with inclusion of questions that invite participants to expand on and/or clarify their accounts [30]. For these reasons, follow-up studies that incorporate other qualitative methods and thus enable a deeper exploration of the comments and reflections offered by participants (e.g. interviews, focus groups) will be necessary to substantiate and broaden the insights we have presented in this article.

Our research is rich in its implications for public health policy and intervention. First, as already noted, some of the preliminary scholarship on the impacts or potential impacts of the COVID-19 context on trans and non-binary people has highlighted isolation and declining mental health as representing key effects of the pandemic on these groups [16, 20]. Our study complicates this empirical picture by revealing that trans and non-binary people may experience poor mental health as a secondary impact of pandemic-related systemic changes to healthcare access, socioeconomic stability, social networks, safety, and gender affirmation. Accordingly, we believe public health policy and intervention addressing the impacts of the COVID-19 context on trans and non-binary people may need to target these domains as potential root causes to mitigate the growing health disparities affecting these groups, including those related to mental health specifically. Despite the presence of policies such as the Canada COVID-19 Economic Response Plan [26], as well as a recognition of the need for poverty alleviation initiatives addressing the issues of LGBTQ2 populations (particularly trans persons) in Canada’s Poverty Reduction Strategy [28], more substantive structural interventions addressing economic security are needed to target these disparities. Although we strongly believe in the necessity of trans-inclusive mental health services to help address the secondary impacts of the pandemic, these need to be designed and delivered alongside measures that reduce discrimination and enhance the provision of trans-affirming healthcare, strengthen employment and housing security, and improve the capacity of informal peer communities to thrive.

A final implication of our research involves leveraging our insights on the positive experiences of some of our respondents in the context of the COVID-19 pandemic. Many participants described finding novel solutions to some of the systemic problems identified in our findings, including the use of virtual healthcare (despite 15.1% of survey respondents indicating that they avoided virtual healthcare or telehealth for fear of experiencing stigma and discrimination [42]), shared housing, and the use of online platforms for community building. Accordingly, we believe public health policymakers and practitioners can draw on these experiences to inform targeted programming to support trans and non-binary populations, including virtual care delivery that is intentionally inviting of trans and non-binary people. For example, practitioners can consider developing virtual platforms, both for the purpose of brokering healthcare resources, and for building peer-led communities of support, to assist with mitigating some of the adverse conditions reported among those in our sample. Policymakers can, similarly, draw on our findings to incentivize peer-led virtual and face-to-face programming for trans and non-binary communities, by creating and promoting funding opportunities for these services. Although these measures would not immediately address the broader systemic effects of the pandemic on trans and non-binary people’s experiences with issues such as healthcare and socioeconomic insecurity, we believe they would enhance community capacity and potentiate change over time. As addressing the far-reaching impacts of the COVID-19 pandemic will likely require long-term commitment to the health and well-being of marginalized populations most prominently affected by this context, we hope our recommendations serve to inform responses to the impacts of the pandemic on trans and non-binary people.

## Conclusions

Research regarding the social conditions and experiences of trans and non-binary people has, prior to the COVID-19 pandemic, revealed that these groups are affected by significant social and health inequities [17, 18]. Given this context, it is perhaps not surprising that our findings revealed significant impacts of the COVID-19 pandemic on these populations in Canada, including an increase in healthcare barriers, socioeconomic precarity, and safety issues, and changes in the social networks and gender affirmation experiences of those in these groups. Recognizing that scholarship on the issues of trans and non-binary people in the COVID-19 context continues to be nascent, we hope that our findings catalyze ongoing inquiry in this area, which may in turn serve to inform public health policy and practice. Though we have outlined preliminary directions in research, policy, and intervention, we believe ongoing research will contribute to the strengthening of evidence concerning the health of trans and non-binary people amid the COVID-19 pandemic, and function to refine our insights on implications of this research for public health.

## Data Availability

All data used for analysis are included as direct participant quotes in the body of the manuscript.

## Abbreviations

NMREB: Non-medical research ethics board
BC: British Columbia
LGBTQ+: Lesbian, gay, bisexual, transgender (trans), queer, and other sexual and gender minority communities
CAD: Canadian dollars
